# Clinical presentation, microbiological profile, and management challenges of infective endocarditis: a retrospective study from a high volume cardiac centre in Pakistan

**DOI:** 10.1186/s43044-025-00699-z

**Published:** 2025-11-10

**Authors:** Muhammad Wali Saleem, Maha Amjad, Ihsan Ullah, Rafi Ullah Jan, Muhammad Ishaq Khan, Ummad Israr

**Affiliations:** 1 Peshawar Institute of Cardiology, Peshawar, Pakistan; 2 Rehman Medical College, Peshawar, Pakistan

**Keywords:** Infective endocarditis, Antibiotic resistance, MACE, Blood culture, Clinical outcomes, Retrospective study

## Abstract

**Background:**

Infective endocarditis (IE) is a potentially fatal condition with high morbidity and mortality. This single center study was designed to assess the clinical presentation, causative organisms, antibiotic resistance, and clinical outcomes of IE in patients treated at a tertiary care cardiac center in Pakistan.

**Methods:**

A retrospective observational study was conducted at a large tertiary care cardiology center in Peshawar, Pakistan from July 2021 to July 2023. Data was collected from hospital records, including demographic, clinical, and laboratory parameters. Statistical analysis was performed using Stata version 14.2.

**Results:**

Among 84 patients, 41.7% were male with a mean population age of 49.17 ± 18.55 years, and an average BMI of 27.72 ± 4.37 kg/m^2^. Hypertension was the most common comorbidity, found in 47.6% patients, followed by diabetes in 36.9% patients. Streptococcus Viridans (25%) was the most common organism isolated, followed closely by Staphylococcus aureus at 22.6%. Surprisingly, 32.1% of the patients had negative cultures. Antibiotic resistance was observed in 25/57 (43.9%) of culture positive cases, and Major Adverse Cardiovascular Events (MACE) occurred in 56%. Acute kidney injury was observed in 48.8% of the patients.

**Conclusion:**

IE presents diverse etiologies and outcomes, necessitating targeted management strategies to reduce antibiotic resistance and improve outcomes in such a challenging subsets of patients.

## Introduction

Infective endocarditis (IE) is a potentially fatal disease process resulting from infection and inflammation of the endocardial surface that most often involves the cardiac valves [1]. It is considered to be the fourth most common life-threatening infection, after pneumonia, sepsis and intra-abdominal infections [2]. Epidemiological data from developed countries estimate the incidence to be about 3 to 7 cases per 100,000 person years with an increasing trend seen in the recent decades [3]. 

IE remains a complex disease with heterogeneous presentations making it challenging to diagnose and treat. It is an infection of the endocardium, typically the heart valve’s lining with a predisposition for patients with preexisting valvular abnormalities, immunodeficiency or history of intravenous drug use. The clinical manifestations can range from low-grade fever and fatigue to heart failure, septic embolism, and stroke [4].

Pathogenesis of IE involves microbial colonization of damaged endocardial surfaces. This subsequently results in the formation vegetations consisting of debris from fibrin, platelets, and microorganisms. These vegetations may break off and cause blockages. This can lead to other complications like infarctions and abscesses [5]. 

Historically, infective endocarditis was seen in people with rheumatic heart disease, especially in developing countries. Nonetheless, with the advances of cardiac surgery and the widespread use of invasive procedures, there is a significant shift in its epidemiology [6]. Endocarditis of prosthetic valves and health-care associated infections related to intravascular devices and immunosuppressive therapy have come forth as important causes in developed countries [7]. The rising incidences of IE occurring in a prosthetic valve, bioprosthetic implants and valve repair surgery highlight the changing risk factors in modern hospital practice [8]. 

Microbiologically Streptococcus viridans and Staphylococcus aureus continue to be the most common organism involved. Although Streptococcus species are typically implicated in subacute presentations associated with dental and oral infections, Staphylococcus aureus is more aggressive and is usually implicated in healthcare-associated infections and intravenous drug use [9]. In recent times, the emergence of multi-drug-resistant organisms like enterococcus and gram-negative bacilli has made treatment more difficult. Due to the changing patterns of microorganisms, we need continuous monitoring and amendments in the antibiotic policy [10]. 

Despite better diagnostic tools (like echocardiography and blood culture techniques) and the treatment (e.g., valve replacement surgery and prolonged intravenous antibiotics), mortality due to IE remains high. A large meta-analysis by Abegaz et al. revealed that IE remains a lethal disease with long-term (1-year and 5-year) mortality of 40% and 70%, respectively. A delayed diagnosis, poor empirical therapy, and excessive presence of multidrug resistant organisms is often associated with poor prognosis [11, 12]. 

In Pakistan, the burden of IE remains poorly characterized with limited epidemiological data available. Lack of clear national guidelines on diagnosis and treatment result in uncertainty in diagnosis and treatment. This study aimed to characterize the profile of IE in the population of Khyber Pakhtunkhwa, Pakistan. The primary objective was to determine the frequency, demographics, clinical characteristics of the affected population and establish common causative microorganism along with rates of antibiotic resistance. Furthermore, the study aimed to analyze the clinical outcomes in terms of Major Adverse Cardiovascular Events (MACE) and other in hospital complications to aid in local and national management prospective.

## Methods

### Study design and setting

This was a retrospective observational study conducted at the Department of Cardiology of a large tertiary care cardiology center in Peshawar, Pakistan. The data collection period spanned two years, from July 2021 to July 2023. The study focused on evaluating the frequency, causative organisms, antibiotic resistance, and clinical outcomes of infective endocarditis (IE) in patients admitted to the tertiary care cardiac center.

### Study population

Patients included in the study were aged between 20 and 80 years and presented with a diagnosis of IE, confirmed using the Modified Duke’s Criteria. Both male and female patients were eligible for inclusion. Patients with native valve, prosthetic valve, or cardiac device-related infective endocarditis were included.

### Inclusion criteria

1. Patients aged 20–80 years.
2. Both male and female patients.
3. Definite and Possible IE cases diagnosed based on Modified Duke’s Criteria.
4. Patients with native valve, prosthetic valve, or cardiac device-related infective endocarditis.

### Exclusion criteria

1. Patients who did not provide consent.
2. Patients classified as rejected IE based on Modified Duke’s Criteria.
3. Patients without available blood culture reports.

### Sampling technique

A non-probability consecutive sampling technique was employed. All eligible patients who met the inclusion criteria and were admitted during the study period were included.

### Data collection procedure

Following approval from the Institutional Ethical Review Board, data was retrieved retrospectively from the hospital’s electronic database, the Hospital Management Information System (HMIS). Each patient’s demographic data, clinical presentation, laboratory parameters, microbiology reports, antibiotic regimens, imaging results, treatment responses, and outcomes were recorded using a structured proforma.

Patients were contacted over the telephone to obtain consent for participation in the study and to verify clinical information where necessary. In cases where the patient was deceased, the next of kin were contacted to obtain consent and clinical data. Data collected included demographic details (age, gender, BMI, and residence), presenting symptoms, comorbidities (diabetes, hypertension, smoking status, history of cancer, immune deficiencies, and previous cerebrovascular accidents), and laboratory investigations (C-reactive protein, total leukocyte count, hemoglobin, platelet count, creatinine, and estimated glomerular filtration rate).

The echocardiographic findings, such as presence of vegetation and the site were recorded. Details of empirical and culture-guided antibiotic regimens, resistance patterns, and outcomes, including Major Adverse Cardiac Events (MACE), were also documented.

### Operational definitions

1. *Infective Endocarditis Diagnosis*: IE was diagnosed based on Modified Duke’s Criteria and classified as definite, possible, or rejected. Definite cases required two major criteria, one major and three minor criteria, or five minor criteria. Possible cases were one major and one minor criterion or three minor criteria.
2. *Major Adverse Cardiovascular Events (MACE)*: Defined as the occurrence of heart failure, pulmonary edema, myocardial infarction, stroke, or cardiac death during hospitalization.
3. *Antibiotic Resistance*: Defined as the presence of non-susceptibility to at least one commonly used antibiotic (penicillin, ceftriaxone, vancomycin or gentamicin) for infective endocarditis based on sensitivity testing.
4. *Negative Blood culture*: No microbiological growth seen in at least 3 sets of cultures taken from different venipuncture sites over 12 h.
5. *Renal Dysfunction*: Acute Kidney Injury (AKI) was defined as a rise in creatinine by ≥ 0.3 mg/dL within 48 h or ≥ 1.5 times the baseline within seven days.
6. *Vegetation on Echocardiography*: Defined as an oscillating intracardiac mass or an area of abnormal echo density attached to a valve, prosthetic material, or endocardial surface.

### Data analysis procedure

Data was entered and analyzed using Stata version 14.2. Continuous variables were assessed for normality using the Shapiro-Wilk test. Normally distributed data were reported as means with standard deviations where as non-normal distribution was displayed as median and interquartile range. Categorical variables were expressed as frequencies and percentages.

Inferential statistics were applied to identify associations between infective endocarditis and clinical outcomes. The Chi-square test or Fisher’s exact test (for small cell counts) was used to assess associations between categorical variables, such as the presence of infective endocarditis and blood culture organisms, antibiotic resistance, and MACE.

A p-value of ≤ 0.05 was considered statistically significant. Results were presented as tables and figures to highlight descriptive and inferential statistics.

### Ethical considerations

The study adhered to ethical principles outlined in the Declaration of Helsinki. Ethical approval was obtained from the Institutional Review Board at MTI-Peshawar Institute of Cardiology prior to data collection. Participants were contacted over the phone, and informed consent was obtained. In cases where the patient was deceased, consent was obtained from the next of kin. The purpose of the study, confidentiality, and the voluntary nature of participation was explained to all participants.

To ensure privacy, patient data was kept anonymous, and all records were securely stored. Participation was voluntary, and patients retained the right to withdraw at any time without any implications on their treatment or care.

### Study limitations

Being a retrospective observational study, findings may be subject to information bias due to reliance on previously recorded data. Additionally, incomplete follow-up data and missing laboratory records may have influenced the analysis of outcomes. Detailed sensitivity and resistance data of individual patients was not recorded, only the presence or absence of resistance as defined by the operational definitions was recorded. Total duration of antibiotic treatment received by individual patients was not included due to incomplete data.

## Results

A total of 84 patients were recruited to the study. The population comprised 41.7% males and 58.3% females. 47.6% of the patients had pre-existing hypertension, while 36.9% had diabetes. Moreover, 28.6% of the patients were smokers, and 21.4% had a history of cerebrovascular accidents (CVA). Intravenous drug abuse (IVDA) was reported in 15.5% of the population. Around 20.2% of patients had prosthetic heart valves whereas the remaining had native valve disease (Table 1).

The mean age of the study participants was 49.17 years with a standard deviation of 18.55 years. The mean BMI was 27.72 ± 4.37 kg/m². The mean duration of symptoms among participants was 14.72 ± 8.73 days while the average hospital stay duration was 18.13 ± 7.35 days, median and interquartile ranges (IQR) in Table 1. Laboratory findings revealed that the mean CRP on arrival was 47.9 ± 28.92 mg/L, total leukocyte count (TLC) was 7846.26 ± 2312.10 cells/µL, and hemoglobin (HB) was 12.22 ± 2.51 g/dL. The mean platelet count on arrival was 297500.54 ± 87484.54 cells/µL. Renal function tests showed a mean creatinine level of 1.21 ± 0.42 mg/dL and an estimated glomerular filtration rate (eGFR) of 91.14 ± 17.82 mL/min/1.73 m² (Table 1).

**Table 1:** Population demographics (*n* = 84)

Variable	Frequency (*n*)	Percentage
Male	35	41.7
Hypertension	40	47.6
Diabetes	31	36.9
Smoking	24	28.6
Prior CVA	18	21.4
Intravenous drug abuse	13	15.5
Native valve	67	79.8
Prosthetic valves	17	20.2

Quantitative variables		
Variable	Mean	Standard deviation
Age (years)	49.17	18.55
BMI (kg/m^2^)	27.72 kg/m²	4.37
CRP on arrival (mg/L)	47.90	28.92
TLC on arrival (cells/µL)	7846.26	2312.10
HB on arrival (g/dL)	12.22	2.51
Platelets on arrival (cells/µL)	297500.54	87484.54
Creatinine on arrival (mg/dl)	1.21	0.42
eGFR on arrival (mL/min/1.73 m²)	91.14	17.82
	*Median*	*IQR*
Duration of symptoms (days)	16.00	16.75
Duration of hospital stay (days)	17.50	14.50

The most common presenting symptom was shortness of breath (40.5%) followed by chest pain (27.4%). Cough and fever had similar frequencies at 16.7% and 15.5% respectively (Table 3). Fever was documented on arrival in 46.4% patients whereas 45.2% patients reported antibiotic use prior to admission. Vegetations were seen on echo of 79.8% patients (Table 2).

**Table 2:** Clinical characteristics (*n* = 84)

Variable	Frequency (*n*)	Percentage
Chest pain	23	27.4
Cough	14	16.7
Fever	13	15.5
Shortness of breath	34	40.5
Documented fever on arrival	39	46.4
Received antibiotics on arrival	38	45.2
Vegetation on echo	67	79.8

**Fig. 1:**
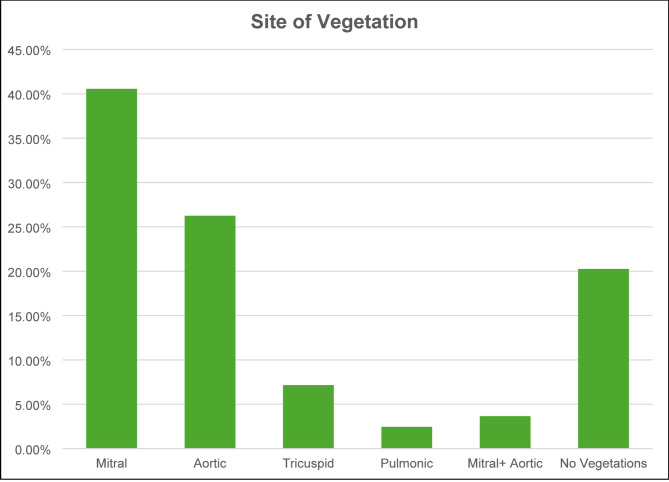
Graphical representation of most common sites of vegetations

The most common site of vegetation was the mitral valve (40.5%) followed by the aortic valve (26.2%). Tricuspid and pulmonic valve involvement was less frequently seen (Fig. 1).

Approximately 32.1% of the blood cultures yielded no growth. The distribution of organisms isolated from blood cultures revealed that Streptococcus Viridans was the most frequently identified pathogen, occurring in 25% of cases. Staphylococcus aureus was the second most common organism, being reported in 22.6% of cases. Coagulase-negative staphylococci were observed in 14.3% of the cultures, and Enterococcus was detected in 6%. Antibiotic resistance was observed in 43.9% of culture positive cases, while 56.1% were susceptible to antibiotics. Based on the Modified Duke’s Criteria, 64.3% of cases were classified as definite infective endocarditis, while 35.7% were classified as possible (Table 3).

**Table 3:** Culture and sensitivity results and DUKES criteria probability (*n* = 84)

	Frequency	Percent
*Organisms in blood*		
Culture negative	27	32.1
Enterococcus	5	6.0
Staphylococcus Aureus	19	22.6
Streptococcus Viridans	21	25.0
Coagulase Negative Staphylococci	12	14.3
Total	84	100.0
*Resistance to antibiotics (culture positive cases)*		
Yes	25	43.9
No	32	56.1
Total	57	100.0
*Probability of infective endocarditis*		
Definite	54	64.3
Possible	30	35.7
Total	84	100.0

**Fig. 2:**
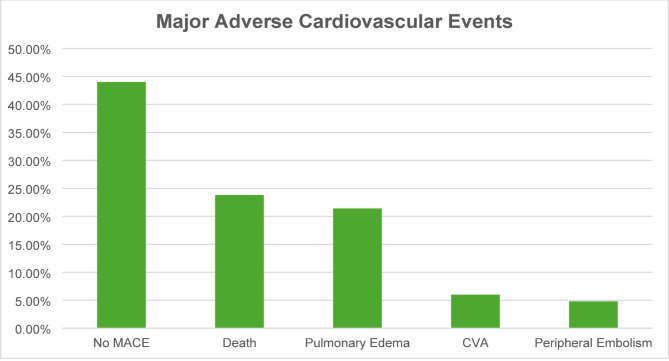
Major adverse cardiovascular events (MACE) during hospitalization

MACE was observed in 56% of the patients, with 23.8% patients dying during admission. Pulmonary edema was observed in 21.4% of the patients, while CVA and peripheral embolism were observed in 6% and 4.8% of the patients, respectively (Fig. 2). Apart from MACE, AKI was observed in 48.8% of the patients during admission. 22 (26.2%) of the patients underwent surgical treatment for infective endocarditis (Table 4).

**Table 4:** Complications during hospitalization (*n* = 84)

Variable	Frequency (*n*)	Percentage
No MACE	37	44.0
Death	20	23.8
Pulmonary edema	18	21.4
CVA	5	6.0
Peripheral embolism	4	4.8
Total	84	100
AKI during admission	41	48.8
Surgery proceeded	22	26.2

Antibiotic resistance was most commonly observed in patients with Enterococcus species infection seen in 3/5 cases (60%), followed by Staphylococcus aureus 9/19 (47.37%) and Coagulase-negative Staphylococci cases (41.67%). The least resistance was observed in Streptococcus Viridans species, with 8 of 21 cases showing antibiotic resistance (38%). However, there was no statistically significant difference between the groups in terms of resistance (Table 5). There was no statistically significant difference between the types of organisms found on prosthetic and native valves, nor was there any association between the type of organism grown and the site of vegetation (Tables 6 and 7).

Analysis of MACE and its relation to antibiotic resistance/ Probability on DUKES Criteria failed to show any statistically significant relation (Tables 8 and 9).

Similarly, no statistically significant relationship was observed between the surgical treatment and site of vegetation, valve type and type of organism (Tables 10, 11 and 12).

**Table 5:** Resistance to antibiotics in positive cultures growths (*n* = 57)

	Resistance to antibiotics		Total	*P* value
	Yes	No		
*Organism in blood culture*				
Enterococcus	3	2	5	0.846
Staphylococcus Aureus	9	10	19	
Streptococcus Viridans	8	13	21	
Coagulase negative staphylococci	5	7	12	
Total	25	32	57	

**Table 6:** Type of organism based on valve type (*n* = 84)

Valve type	Enterococcus *n* (%)	Staph. Aureus *n* (%)	Viridans Strep. *n* (%)	Coagulase negative Staph. *n* (%)	Culture Negative *n* (%)	Total	*P* value
Native valve	3 (60.0%)	13 (68.4%)	15 (71.4%)	10 (83.3%)	26 (96.3%)	67 (79.8%)	0.081
Prosthetic valve	2 (40.0%)	6 (31.6%)	6 (28.6%)	2 (16.7%)	1 (3.7%)	17 (20.2%)	
Total	5 (100.0%)	19 (100.0%)	21 (100.0%)	12 (100.0%)	27 (100.0%)	84 (100.0%)	

**Table 7:** Relation between site of vegetation and organism grown on culture (*n* = 84)

Site of vegetation	Enterococcus *n*(%)	Staphylococcus Aureus *n* (%)	Viridans Streptococcus *n* (%)	Coagulase negative Staphylococci *n* (%)	Culture Negative *n* (%)	Total	*P* value
Mitral	2 (40.0%)	8 (42.1%)	10 (47.6%)	4 (33.3%)	10 (37.0%)	34 (40.5%)	0.461
Aortic	2 (40.0%)	3 (15.8%)	3 (14.3%)	7 (58.3%)	7 (25.9%)	22 (26.2%)	
Tricuspid	0 (0.0%)	1 (5.3%)	1 (4.8%)	0 (0.0%)	4 (14.8%)	6 (7.1%)	
Pulmonic	0 (0.0%)	1 (5.3%)	0 (0.0%)	0 (0.0%)	1 (3.7%)	2 (2.4%)	
Mitral + aortic	0 (0.0%)	2 (10.5%)	0 (0.0%)	0 (0.0%)	1 (3.7%)	3 (3.6%)	
No vegetations	1 (20.0%)	4 (21.1%)	7 (33.3%)	1 (8.3%)	4 (14.8%)	17 (20.2%)	
Total	5 (100.0%)	19 (100.0%)	21 (100.0%)	12 (100.0%)	27 (100.0%)	84 (100.0%)	

**Table 8:** MACE and relation to organisms (*n* = 84)

	Organism in blood culture					Total n (%)	P value
	Enterococcus n (%)	Culture negative n (%)	Staphylococcus Aureus n (%)	Streptococcus Viridans n (%)	Coagulase negative staphylococci n (%)		
*MACE during hospital stay*							
No	3 (3.57)	14 (16.67)	6 (7.14)	10 (11.9)	4 (4.76)	37 (44.05)	0.591
Death	2 (2.38)	6 (7.14)	4 (4.76)	4 (4.76)	4 (4.76)	20 (23.81)	
Pulmonary edema	0 (0)	5 (5.95)	7 (8.33)	5 (5.95)	1 (1.19)	18 (21.43)	
CVA	0 (0)	0 (0)	2 (2.38)	1 (1.19)	2 (2.38)	5 (5.95)	
Peripheral embolism	0 (0)	2 (2.38)	0 (0)	1 (1.19)	1 (1.19)	4 (4.76)	
Total	5 (5.95)	27 (32.14)	19 (22.61)	21 (25)	12 (14.28)	84 (100)	

**Table 9:** Relation of MACE to probability on DUKES criteria (*n* = 84)

	Probability on DUKES criteria		Total n (%)	P-value
	Definite n (%)	Possible n (%)		
*MACE during hospital stay*				
No	24 (28.57)	13 (15.47)	37 (44.05)	0.137
Death	9 (10.71)	11 (13.09)	20 (23.81)	
Pulmonary edema	15 (17.86)	3 (3.57)	18 (21.43)	
CVA	4 (4.76)	1 (1.19)	5 (5.95)	
Peripheral embolism	2 (2.38)	2 (2.38)	4 (4.76)	
Total	54 (64.3)	30 (35.7)	84 (100)	

**Table 10:** Relation of surgical treatment to site of vegetation (*n* = 84)

Site of vegetation	Surgery proceeded		Total (n(%)	P value
	Yes n(%)	No n(%)		
Mitral	12 (35.3%)	22 (64.7%)	34 (40.5%)	0.083
Aortic	8 (36.4%)	14 (63.6%)	22 (26.2%)	
Tricuspid	1 (16.7%)	5 (83.3%)	6 (7.1%)	
Pulmonic	0 (0%)	2 (100%)	2 (2.4%)	
Mitral + aortic	1 (33.3%)	2 (66.7%)	3 (3.6%)	
No vegetations	0 (0%)	17 (100%)	17 (20.2%)	
Total	22 (26.2%)	62 (73.8%)	84 (100%)	

**Table 11:** Relation of type of valve and surgical treatment (*n* = 84)

Valve type	Surgery proceeded		Total n (%)	P value
	Yes n (%)	No n (%)		
Native valve	16 (23.9%)	51 (76.1%)	67 (79.8%)	0.91
Prosthetic valve	6 (35.3%)	11 (64.7%)	17 (20.2%)	
Total	22 (26.2%)	62 (73.8%)	84 (100%)	

**Table 12:** Relation of type of organism to surgical treatment (*n* = 84)

Organism in blood culture	Surgery proceeded		Total n (%)	P value
	Yes n (%)	No n (%)		
Enterococcus	1 (20.0%)	4 (80.0%)	5 (6.0%)	0.976
Culture Negative	7 (25.9%)	20 (74.1%)	27 (32.1%)	
Staphylococcus Aureus	5 (26.3%)	14 (73.7%)	19 (22.6%)	
Streptococcus Viridans	5 (23.8%)	16 (76.2%)	21 (25.0%)	
Coagulase Negative Staph	4 (33.3%)	8 (66.7%)	12 (14.3%)	
Total	22 (26.2%)	62 (73.8%)	84 (100%)	

## Discussion

This study provides contemporary insights into the clinical profile, microbiological patterns, antibiotic resistance and outcome of infective endocarditis patients in the population of Khyber Pakhtunkhwa, Pakistan. Our findings reveal key differences compared to global data highlighting younger age of presentation with a mean population age of 49.17 ± 18.55 years, notably higher rates of antibiotics resistance (43.9%) and considerably higher MACE (56%) drawing attention to the need for region specific strategies in diagnosis and management in low middle income countries (LMIC).

The mean age of our study was 49.17 years (SD 18.55 years) which is considerably younger than high-income countries (≥ 60 years) [2, 11, 13]. Similar trends have been observed in other LMIC with a high prevalence of rheumatic heart disease (RHD), untreated valvular disease and poor socioeconomic conditions [5, 14]. 

Hypertension and diabetes remained common comorbidities in our population which is consistent with existing data linking metabolic syndromes to higher cardiovascular co-morbidity [1]. Intravenous drug abuse (IVDA) on the other hand was seen in 15.5% of the population, a rate lower than that seen in Western cohorts where IVDA remains a major risk factor for IE [2, 15]. 

Streptococcus viridans (25%) and staphylococcus aureus (22.6%) remained the predominant causative organisms which is in line with previous data from Pakistan and other LMICs [5, 16]. Culture negative endocarditis however was observed in 32.1% of patients which is much higher than observed in Western studies which vary from 5 to 20% [2, 13]. Contributing factors for this can include prior antibiotic exposure (which was observed in nearly half of our patients), fastidious organism growth that’s not detected by conventional techniques or due to inadequate culture volumes. Culture techniques used at our hospital include Bactec Plus™ (Becton-Dickinson, NJ, USA) systems for incubation with prolonged incubation for cases with suspected fastidious organism growth. Prolonged incubation and subculturing however doesn’t necessarily yield significant microorganism growth in standard automated cultures [17]. Molecular diagnostic techniques like PCR and next-generation sequencing can improve diagnostic yield in culture negative cases but remain under-utilized in LMICs [18]. 

Antibiotic resistance to at least one commonly used empirical antibiotic was 43.9% of culture positive cases raising concerns over the use of empirical antibiotics. These finding corroborate regional data on high multi drug resistance [14, 16, 19]. This is in part due to absence of standardized national guidelines on antibiotic prescribing and limited stewardship of antibiotic accessibility in Pakistan which is likely to exacerbate antibiotic resistance rates. Tailored antimicrobial policies, prescribing guidelines and antibiotic dispensing surveillance are essential to control this serious issue.

MACE occurred in 56%of the patients with an in-hospital mortality in almost a quarter of all admitted patients. These rates are higher than observed in developed countries. An example is the EURO-ENDO registry where the in-hospital mortality is estimated to be around 17.1% in 2019 [13, 20]. AKI was observed in 48.8% of our cohort which is consistent with global data identifying renal dysfunction as a predictor of poor clinical outcomes [21, 22]. 

Surgical intervention was performed in 26% of cases, a rate lower than international standards [22, 23]. One of the major reasons for this in our setting was late referral and financial limitation. Certain surgical interventions including valve replacement were not covered under the National Health insurance leading to limited surgical options. This in turn could have also contributed to a higher in-hospital mortality. Given that timely surgical intervention is associated with survival benefit, these pathways need to be strengthened for better clinical outcomes [23, 24]. 

Our findings align with existing regional studies from Pakistan and other LMICs, with a younger population, higher culture negative cases and significant antibiotic resistance [5, 14, 16, 19]. However, the higher mortality and morbidity rates compared to the Western Population suggest significant room for improvement in terms of diagnostic infrastructure, referral pathways, funding sources, treatment guidelines and population education in general.

The strengths of this study include its focus on a large cardiac referral center with detailed analysis of microbiological and clinical outcomes. Limitations however include the retrospective single center design, absence of long term follow up, lack of detailed resistance profile analysis (e.g., MRSA, VRE) and duration of antibiotic therapy. Moreover, sample size in our study may not be large enough to derive statistically significant inferences. Further studies focusing on the detailed resistance and antibiotic susceptibility as well as the culture techniques employed in LMICs would aid in improving existing literature and standard of care.

## Conclusion

In summary, the clinical and microbiological profile of infective endocarditis in a developing country is varied. The results highlight the importance of tailored diagnostic and therapeutic measures to combat the rising antibiotic resistance and unfavorable results. Identifying the organisms early and treating them specifically and monitoring closely can improve prognosis. Future studies should refine the diagnostic criteria, investigate patterns of resistance and susceptibility, evaluate long-term treatment outcomes and develop region-specific management protocols for this high-risk condition.

## Data Availability

The data that support the findings of this study are available on request from the corresponding author. The data are not publicly available due to privacy or ethical restrictions.

## Abbreviations

IE: Infective endocarditis
MACE: Major Adverse Cardiovascular Events
RHD: Rheumatic heart disease
RF: Rheumatic fever
HMIS: Hospital Management Information System
AK!: Acute Kidney Injury
CVA: Cerebrovascular accidents
IVDA: Intravenous drug abuse
LMIC: Low middle income countries
TLC: Total leukocyte count
HB: Hemoglobin
eGFR: Estimated glomerular filtration rate
